# Estimating mean circulatory filling pressure in clinical practice: a systematic review comparing three bedside methods in the critically ill

**DOI:** 10.1186/s13613-018-0418-2

**Published:** 2018-06-20

**Authors:** Marije Wijnberge, Daniko P. Sindhunata, Michael R. Pinsky, Alexander P. Vlaar, Else Ouweneel, Jos R. Jansen, Denise P. Veelo, Bart F. Geerts

**Affiliations:** 1 Department of Anesthesiology, Academic Medical Center, Amsterdam, The Netherlands; 2 Department of Intensive Care, Academic Medical Center, Amsterdam, The Netherlands; 3 Laboratory of Experimental Intensive Care and Anesthesiology, Academic Medical Center, Amsterdam, The Netherlands; 4 Department of Critical Care Medicine, University of Pittsburgh Medical Center, 1215.4 Lillian S. Kaufmann Bldg, 3471 Fifth Avenue, Pittsburgh, PA 15213 USA; 5 Department of Intensive Care Medicine, Leiden University Medical Center, Leiden, The Netherlands

**Keywords:** Blood pressure, Venous pressure, Blood volume, Intensive care, Critical care, Hemodynamics

## Abstract

The bedside hemodynamic assessment of the critically ill remains challenging since blood volume, arterial–venous interaction and compliance are not measured directly. Mean circulatory filling pressure (*P*_mcf_) is the blood pressure throughout the vascular system at zero flow. Animal studies have shown *P*_mcf_ provides information on vascular compliance, volume responsiveness and enables the calculation of stressed volume. It is now possible to measure *P*_mcf_ at the bedside. We performed a systematic review of the current *P*_mcf_ measurement techniques and compared their clinical applicability, precision, accuracy and limitations. A comprehensive search strategy was performed in PubMed, Embase and the Cochrane databases. Studies measuring *P*_mcf_ in heart-beating patients at the bedside were included. Data were extracted from the articles into predefined forms. Quality assessment was based on the Newcastle–Ottawa Scale for cohort studies. A total of 17 prospective cohort studies were included. Three techniques were described: *P*_mcf_ hold, based on inspiratory hold-derived venous return curves, *P*_mcf_ arm, based on arterial and venous pressure equilibration in the arm as a model for the entire circulation, and *P*_mcf_ analogue, based on a Guytonian mathematical model of the circulation. The included studies show *P*_mcf_ to accurately follow intravascular fluid administration and vascular compliance following drug-induced hemodynamic changes. Bedside *P*_mcf_ measures allow for more direct assessment of circulating blood volume, venous return and compliance. However, studies are needed to determine normative *P*_mcf_ values and their expected changes to therapies if they are to be used to guide clinical practice.

**Electronic supplementary material:**

The online version of this article (10.1186/s13613-018-0418-2) contains supplementary material, which is available to authorized users.

## Background

It is difficult to determine the cause for hemodynamic instability in patients and to predict the best treatments. Currently, cardiovascular resuscitation options are triggered by arterial pressure and cardiac output (CO) measures, focusing on the oxygen delivery side of the circulation. However, the primary determinants of CO reside on the venous side. Veins are 30–50 times more compliant than arteries and contain approximately 75% of the total blood volume [1–5]. Mean circulatory filling pressure (*P*_mcf_) provides vital information on this “forgotten venous side of the circulation” [6].

**Fig. 1:**
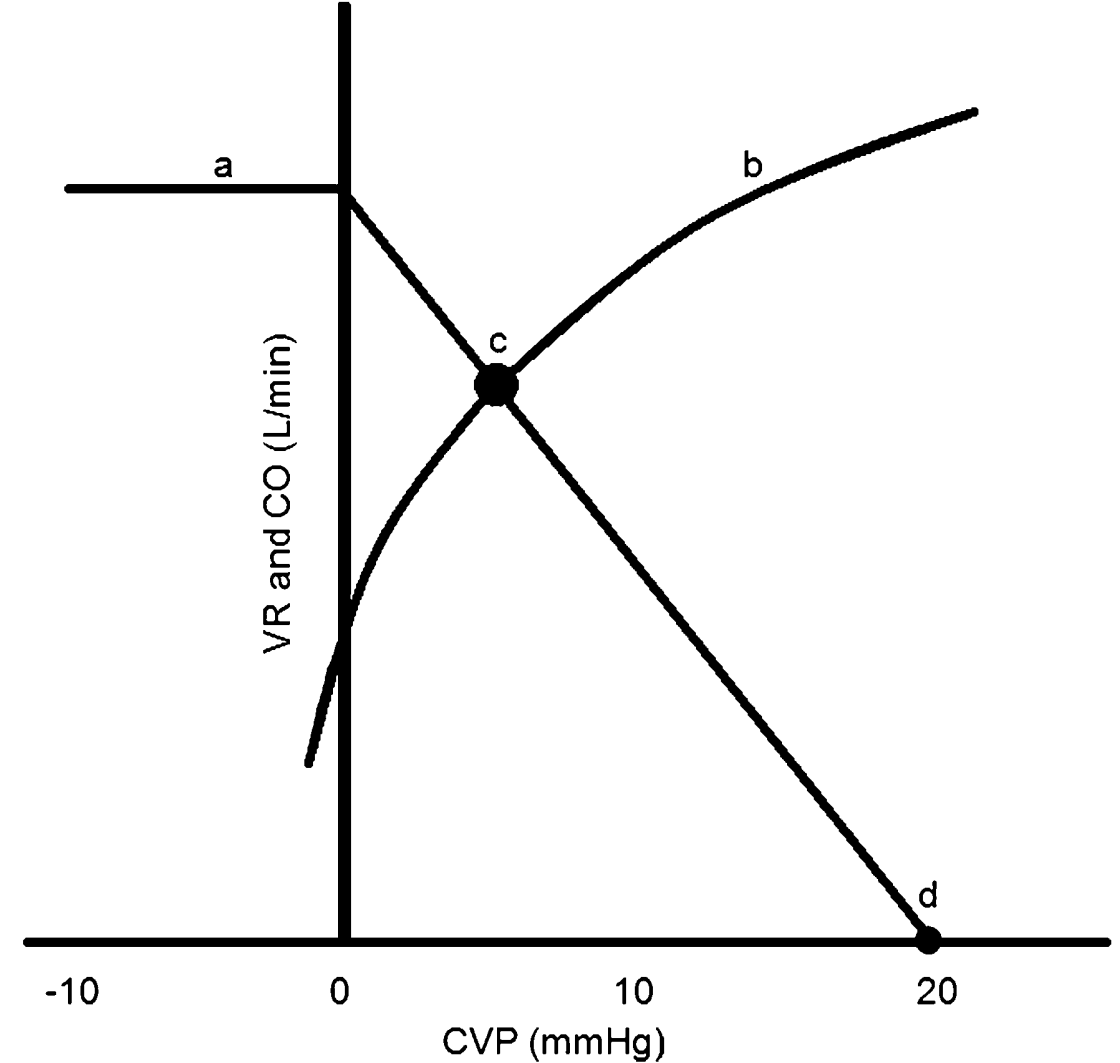
The venous return curve (*a*) combined with the cardiac output curve (*b*). The intersection of these two curves (*c*) is the working point of the circulation. The central venous pressure when venous return equals zero is the *P*_mcf_ (*d*). The slope of the *V*_R_ is determined by the resistance to venous return

In 1894, *P*_mcf_ was defined as the equilibrium pressure throughout the circulation during circulatory arrest [7]. In the 1950s, Guyton and colleagues described a linear relationship between venous return (*V*_R_) and right atrial pressure (*P*_ra_), described as: *V*_R_ = (*P*_mcf_ − *P*_ra_)/(RVR) [8, 9]. RVR is resistance to *V*_R_ and defines the slope of the *V*_R_ curve. This linearity has been confirmed in intact circulations in animal studies and is not affected by hypo- or hypervolemia [10–15]. *V*_R_ curves enable to determine the equilibrium point of the circulation, which is the intersection between the CO and *V*_R_ curve. Central venous pressure (CVP) is a surrogate of *P*_ra_ used in clinical practice. CVP at zero flow equals *P*_mcf_ (Fig. 1).

Vascular volume requires a minimal volume before its distending pressure becomes positive. The amount of blood not causing pressure on the vessels is called unstressed volume (*V*_u_) and reflects intravascular volume present with *P*_mcf_ of zero. Stressed volume (*V*_s_) is the additional blood causing a distending pressure on the vascular walls and reflects the effective circulating volume. *V*_u_ and *V*_s_ together define the total blood volume. *V*_s_ is approximately 25% of the total blood volume [3–5]. *V*_s_ and vascular compliance (Csys) define *P*_mcf_ [16]. An increase in *V*_s_ increases *P*_mcf_, and an increase in Csys decreases *P*_mcf_. Fluid loading should increase *P*_mcf_, but *V*_R_ only increases if the pressure gradient for *V*_R_ (i.e., *P*_mcf_ CVP) increases, RVR decreases, or both. Since in the steady state *V*_R_ = CO, knowing the determinants of *V*_R_ is relevant to understanding cardiovascular state.

Recently, methods have emerged to enable clinicians to estimate *P*_mcf_ at the bedside. Our objectives for this review were to describe the techniques and to highlight their clinical applicability, precision, accuracy and limitations in critically ill patients.

## Materials and methods

### Publication selection

This review was performed according to PRISMA guidelines [17] (Additional file 1) and methodology outlined in the Cochrane Handbook for systematic reviews [18]. No study protocol was published. A PubMed, Embase and Cochrane Library database search was performed with help of a clinical librarian with no restriction on publication date. The search was performed up to May 18, 2017. The search strategy combined the following concepts: (1) “mean systemic filling pressure” or “mean circulatory filling pressure” or “static filling pressure” and (2) “intensive care” or “critical care” or “perioperative” or “intraoperative” (Additional file 1). Titles, abstracts and full-texts were independently screened by two reviewers for relevance (MW and DPS), and discrepancies were resolved by a third reviewer (BFG). The references of the selected articles were examined for additional eligible articles. Studies were included when available in English and full-text, described prospective studies in which *P*_mcf_ estimation methods were examined in heart-beating ICU patients and contained a description of their clinical applicability, precision and accuracy or limitations.

### Data extraction and analysis

Data were extracted into predefined forms. No additional analyses were performed. Critical appraisal was based on the Newcastle–Ottawa Scale for cohort studies [19] to assess the quality of non-randomized studies at study level. A modified version of the scale was used since only five out of nine questions were applicable, resulting in a possible highest score of five stars (Additional file 1).

## Results

### Study selection and characteristics

The initial search identified 369 articles, of which 300 were excluded after screening title and abstract. A total of 53 articles were excluded based on full-text. Two relevant articles were found by citation tracking. Consequently, 17 prospective cohort studies estimating *P*_mcf_ in heart-beating ICU patients were included (Additional file 1). Three different bedside measurement techniques were found. Eight studies estimated *P*_mcf_ applying inspiratory hold maneuvers (*P*_mcf_ hold), three studies during a circulatory stop-flow in the arm (*P*_mcf_ arm) and four studies using a mathematical algorithm (*P*_mcf_ analogue). Two studies compared multiple techniques.

Eleven studies were performed in postoperative cardiac surgery patients (Table 1). All patients were hemodynamically stable without alteration in vasopressor use or fluid therapy during the study protocol. All patients were sedated and mechanically ventilated. In one study, spontaneous breathing efforts were observed [20]. The number of included patients ranged from nine to 80. In all studies, CVP was measured via a catheter in the right internal jugular vein. CO measurement techniques differed between studies (Additional file 1).

**Table 1:** Baseline characteristics for included studies

References	Method	*N*	Patient population (all adult ICU patients)	Age	Male	Timeframe *P*_mcf_ measurement
Maas et al. [21]	*P*_mcf_ hold	12	Postoperative cardiac surgery	64 (10)	10 (83%)	Not described
			10 CABG			
			2 AVR			
Keller et al. [23]	*P*_mcf_ hold	9	Postoperative cardiac surgery	Median 61	4 (44%)	Not described
			3 CABG	IQR 55–75		
			6 AVR			
Maas et al. [22]	*P*_mcf_ hold	10	Postoperative cardiac surgery	64 (11)	9 (90%)	Within 1 h after ICU admission
			2 AVR			
			1 MVP + TVP			
			7 CABG			
Persichini et al. [27]	*P*_mcf_ hold	16	Septic shock	67 (16)	8 (50%)	Not described
Maas et al. [25]	*P*_mcf_ hold	16	Postoperative cardiac surgery	64 (11)	Not described	Within 1 h after ICU admission
			1 MVP			
			15 CABG			
Guerin et al. [28]	*P*_mcf_ hold	30	Shock	65 (12)	21 (70%)	Not described
De Wit et al. [24]	*P*_mcf_ hold	17	Postsurgical gastrointestinal	62 (9)	14 (82%)	Not mentioned
			16 esophageal resection			
			1 pancreaticoduodenectomy			
Helmerhorst et al. [26]	*P*_mcf_ hold	22	Postoperative cardiac surgery	63 (59–66)	17 (85%)	1 h after ICU admission
			22 CABG			
Geerts et al. [43]	*P*_mcf_ arm	24	Postoperative cardiac surgery	64 (10)	19 (79%)	Within 2 h after ICU admission
			17 CABG			
			7 CABG plus valve repair			
Aya et al. [41]	*P*_mcf_ arm	20	Postoperative cardiac surgery	63 (11)	17 (85%)	Initial period at ICU (not further defined)
			13 CABG			
			4 AVR			
			4 MVR			
Aya et al. [42]	*P*_mcf_ arm	80	Postoperative cardiac surgery	70	62 (78%)	Initial period at ICU (not further defined)
			36 CABG	Range 52–80		
			27 AVR + CABG			
			12 MVR + CABG			
			5 Other			
Parkin et al. [49]	*P*_mcf_ analogue	10	Multi-organ failing patients receiving CVVH for acute renal failure	65	7 (70%)	Not described
				Range 24–77		
Cecconi et al. [48]	*P*_mcf_ analogue	39	22 Cardiac surgery	68 (12)	26 (67%)	Not described
			8 Shock			
			6 Non-cardiac surgery			
			3 Other			
Gupta et al. [20]	*P*_mcf_ analogue	61	Postoperative cardiac surgery	63 (11)	46 (75%)	Within 6 h after ICU admission
			40 CABG			
			8 CABG + valve replacement			
			8 Valve replacement			
			5 Bentall’s procedure			
			7 DDD pacing			
Aya et al. [51]	*P*_mcf_ analogue	26	Postoperative fluid challenge	68	16 (62%)	Initial period at ICU (not further defined)
			7 Cardiac surgery	Range 53–80		
			19 Non-cardiac surgery			
Maas et al. [16]	*P*_mcf_ hold	11	Postoperative cardiac surgery	64	9 (82%)	Within 2 h after ICU admission
	*P*_mcf_ arm	11	9 CABG	Range 50–80		
	*P*_mcf_ analogue	11	2 AVR			
Maas et al. [30]	*P*_mcf_ arm	15	Postoperative cardiac surgery	64 (11)	Not described	Within 1 h after ICU admission
	*P*_mcf_ hold	12	9 CABG			
			5 Valve			
			1 CABG + valve			

Age is presented as mean with standard deviation (SD) or median with range or interquartile range (IQR). Number of males per study is presented as counts with percentage

*CABG* coronary artery bypass, *MVR* mitral valve replacement, *MPV* mitral valve prolapse, *AVR* aortic valve replacement, *TVP* tricuspid valve prolapse, *CVVH* continuous veno-venous hemodiafiltration

## *P*_mcf_ hold

### Technique description

*P*_mcf_ hold is based on the linear relation between CVP and *V*_R_ (*P*_mcf_ = (*V*_R_ − CVP)/RVR). CVP is raised by performing a series of end-inspiratory hold maneuvers. In 2009, the method was first studied in humans [21]. Inspiratory hold maneuvers at 5, 15, 25 and 35 cmH_2_O incremental ventilatory plateau pressures (*P*_vent_) were performed, and CO was measured in the last 3 s of the 12 s inspiratory hold. They validated that after 7–10 s a steady state consists when *V*_R_ = CO. By plotting the CVP and CO values, a *V*_R_ curve is constructed and the zero-flow pressure (*P*_mcf_) extrapolated. Seven studies [16, 21–26] estimated *P*_mcf_ hold using these four plateau pressures. Two studies [27, 28] used two points (*P*_vent_ 5 and 30 cmH_2_O) at 15-s inspiratory and expiratory hold plateau phase. Between the *P*_mcf_ hold measurements, either 1-min pauses were used to re-establish the initial hemodynamic steady state [16, 21, 22, 24, 28], or the consecutive inspiratory hold was performed when CO had returned to baseline [23, 26, 27].

### Clinical applicability

The average baseline *P*_mcf_ hold values found in the eight included studies range from 19 to 33 mmHg with a wide standard deviation (Tables 2, 3). Five studies [21–23, 26, 28] demonstrated fluid administration caused an increase in *P*_mcf_ hold, confirming that in humans, as in animals before [14, 15], *P*_mcf_ hold follows hemodynamic changes (Table 2). One of these studies found passive leg raising (PLR) to significantly increase *P*_mcf_ hold values [28]. RVR was not significantly affected by different volumetric conditions nor by PLR. *V*_s_ was calculated from *P*_mcf_ as a measure for effective circulating volume [22]. In one study, *P*_mcf_ was used to assess the hemodynamic effects of arterial hyperoxia (FiO_2_ = 90% for 15 min) in ICU patients [26]. During this hyperoxia, left ventricular afterload increased and contractility remained similar; however, CO did not decrease. Both *P*_mcf_ and RVR increased significantly (Table 3), explaining why *V*_R_ (thus CO) remained unaltered.

**Table 2:** Mean circulatory filling pressure during different volumetric state

Study	Method	*N*	Patient population	Baseline position	Baseline *P*_mcf_	Hypervolemia (induced by fluid administration)	*p* value*	Amount of fluid administered to induce hypervolemia	Hypovolemia (induced by HUT)	*p* value^†^
Maas et al. [21]	*P*_mcf_ hold	12	Cardiac surgery	Supine	18.7 (4.5)	29.1 (5.2)	0.001	500 mL colloid in 15–20 min	14.5 (3.0)	0.005
Keller et al. [23]	*P*_mcf_ hold	9	Cardiac surgery	Semirecumbent	19.7	26.9	< 0.05	500 mL colloid	–	–
					IQR 17.0–22.6	IQR 18.4–31.0				
Maas et al. [22]	*P*_mcf_ hold	10	Cardiac surgery	Not described	18.7 (4.0)	26.4 (3.2)	< 0.001	500 mL colloid	–	–
Guerin et al. [28]	*P*_mcf_ hold	30	Shock	Semirecumbent	Responder: 25 (13)	32 (17)	< 0.01	500 mL saline in 10 min		
					Non-responders: 24 (10)	28 (12)	< 0.01			
Geerts et al. [43]	*P*_mcf_ arm	24	Cardiac surgery	Supine	Responders: 16.2 (6.3)	22.0 (7.6)	< 0.001	500 mL colloid	–	–
					Non-responders: 24.3 (8.2)	29.9 (9.1)	< 0.001			
Aya et al. [41]	*P*_mcf_ arm	20	Cardiac surgery	Supine	22.4 (7.7)	–	–	–	–	–
Aya et al. [42]	*P*_mcf_ arm	80	Cardiac surgery	Supine	23.0	–	–	–	–	–
					Range: 17.3–29.8					
Parkin et al. [49]	*P*_mcf_ analogue	10	CVVH	Not described	Target state = 15.9	–	–	CVVHD	–	–
Cecconi et al. [48]	*P*_mcf_ analogue	39	Heterogenous	Not described	Responders: 17.8 (5.1)	20.9 (5.1)	< 0.001	Mean 252 (8.9) mL	–	–
					Non-responders: 17.9 (5.1)	21.0 (4.9)	< 0.001	52.5% crystalloid		
								37.6% colloid		
								8.8% FFP & RBC		
Gupta et al. [20]	*P*_mcf_ analogue	61	Cardiac surgery	Supine	Responders: 17 (3.7)	19 (4.3)	0.02	Mean 264 (16) mL	–	–
					Non-responders: 17 (3.6)	19 (4.1)	0.03	50% saline. Other 50%: mix of FFP, platelets, albumin, packed RBC, return of pump blood		
Aya et al. [51]	*P*_mcf_ analogue	26	Heterogenous	Not described	Responders: 13.7 IQR: 10.9–16.9			250 mL crystalloid		
					Non-responders: 16.7 IQR: 10.5–18.9					
Maas et al. [16]	*P*_mcf_ hold	11	Cardiac surgery	Supine	19.7 (3.9)	28.3 (3.6)	< 0.001	500 mL colloid	16.2 (3.0)	0.001
	*P*_mcf_ arm				18.4 (3.7)	27.1 (4.0)	< 0.001		15.4 (3.1)	0.001
	*P*_mcf_ analogue				14.7 (2.7)	19.2 (1.1)	< 0.001		10.9 (2.0)	< 0.001
Maas et al. [30]	*P*_mcf_ arm	15	Cardiac surgery	Supine	21.0 (6.8)	27.7 (7.4)	< 0.001	500 mL colloid (10 steps of 50 mL)	–	–
	*P*_mcf_ hold^#^									

Data presented as mean with SD or median with interquartile range (IQR). *P*_mcf_ in mmHg. Hypovolemic state induced by head up tilt (HUT) to 30°. Responders = fluid responsiveness was defined by a 10% increase in CO

* *p* value, difference between baseline and hypervolemia induced by fluid administration

^†^*p* value, difference between baseline and hypovolemic state

**Table 3:** *P*_mcf_ and pharmacodynamics

References	Method	*n*	Situation A	Situation B	*p* value*	Situation C	*p* value^#^
Persichini et al. [27]		16	NE 0.30	NE 0.19			
			Range 0.10–1.40	Range 0.08–1.15			
	*P*_mcf_ hold (in mmHg)		33 (12)	26 (10)	0.003		
Maas et al. [25]		16	Baseline 1	NE increase of 0.04 (0.02)		Baseline 2	
			NE 0.04 (0.03)			NE 0.04 (0.03)	
	*P*_mcf_ hold (in mmHg)		21.4 (6.1)	27.6 (7.4)	< 0.001	22.0 (5.3)	
de Wit et al. [24]		17	Propofol low	Propofol medium		Propofol high	
			Cb 3.0 (0.90) μg/mL	Cb 4.5 (1.0) μg/mL		Cb 6.5 (1.2) μg/mL	
	*P*_mcf_ hold (in mmHg)		27.9 (5.4)	24.6 (4.9)	0.01	21.4 (4.2)	< 0.001
Helmerhorst et al. [26]		22	FiO_2_ 21–30%	FiO_2_ 90%			
	*P*_mcf_ hold (in mmHg)		20.8 (3.5)	23.1 (4.0)	< 0.001		

*NE* norepinephrine dose in μg/kg/min presented as mean with range or mean with standard deviation. *P*_mcf_ values are presented as mean with standard deviation. *Cb* target blood concentration of propofol in μg/mL. *P*_mcf_ hold values presented in mmHg. *FiO*_*2*_ fractional oxygen concentration

* *p* value, *p* value for situation A compared to B

^#^*p* value, *p* value for situation A compared to C

Studies have used *P*_mcf_ hold to describe hemodynamic changes caused by propofol [24] and norepinephrine [25, 27] (Table 3). In septic shock patients, decreasing the dose of norepinephrine decreased both *P*_mcf_ and RVR [27]. Further, after increasing norepinephrine CO decreased in ten patients and CO increased in six patients [25]. In all patients, *P*_mcf_ and RVR increased, though the “balance” between the two values determined whether CO increased. One study showed an increase in propofol caused a decrease in *V*_s_ without a change in CO [24]. These studies show *P*_mcf_ behaves within the framework of hemodynamic reasoning and lends itself to being used as a less invasive method to assess drug-induced physiology. Since *P*_mcf_ exists at the intersection of arterial and venous flow, it enables to calculate the true arterial and venous resistance by calculating the critical closing pressure (*P*_cc_). *P*_cc_ is the mean arterial pressure (MAP) to zero CO-intercept. Arterial resistance is calculated as (MAP − *P*_cc_)/CO [22].

### Precision and accuracy

The technique precision has not yet been assessed in humans. However, in an animal study the averaged coefficient of variation for repeated measurements of *P*_mcf_ hold was 6% [29]. Comparing the techniques’ accuracy, no significant differences between *P*_mcf_ hold and *P*_mcf_ arm existed, whereas *P*_mcf_ analogue values were significantly lower [16, 30].

### Limitations

The use of *P*_mcf_ hold is restricted to mechanically ventilated and sedated patients with a central venous catheter. The procedure of the inspiratory hold maneuvers is not yet automated and requires a direct link between monitor and ventilator, or advanced monitor analytics to detect the inspiratory holds and to perform the instantaneous CO calculations. Furthermore, it is not suitable during cardiac arrhythmia. This method is not suitable to measure rapid changes in hemodynamic status since it takes a couple of minutes to perform the multiple end-inspiratory (and end-expiratory) holds. Potentially, this technique is operator-dependent because a proper inspiratory plateau pressure is needed. CVP can be altered due to incorrect catheter placement. An absolute CO value is not necessary for *P*_mcf_ hold as the technique extrapolates to zero CO. If the trend measurements are accurate, the RVR slope might change, but the intersection *P*_mcf_ point remains constant. The latter holds only true for the *P*_mcf_ itself, the RVR is dependent of the slope of the curve. In clinical practice, a physician would use *P*_mcf_ together with RVR; therefore, for clinical use of the *P*_mcf_ an accurate CO value is needed.

Potentially, the inspiratory hold maneuver overestimates *P*_mcf_ by the blood translocation from the pulmonary into the systemic circulation [31–33]. However, the potential volume shifts relative to Csys suggest that this effect is minimal [10, 34]. During inspiratory hold maneuvers, arterial pressure decreases. If sustained, baroreflex-induced increased sympathetic tone may cause *P*_mcf_ to increase [35, 36]. Indeed one study performed in pigs found the *P*_mcf_ hold overestimating compared to a method using right atrial balloon occlusion in euvolemic conditions, in bleeding and hypervolemia; however, the values found between the two methods were similar [34]. Two clinical studies [16, 30] have shown *P*_mcf_ hold and *P*_mcf_ arm values not being significantly different, debating the former result found in pigs. Future studies in humans are needed. Moreover, all patients undergoing inspiratory holds are on neuro-humoral suppressive agents, probably dampening the baroreflex and other autonomic influences [37–39].

## *P*_mcf_ arm

### Technique description

As *P*_mcf_ is defined as the steady-state blood pressure during no-flow conditions, instantaneously *P*_mcf_ should mainly be similar for different vascular compartments even though each compartment may have different *V*_u_ and *V*_s_ [2, 40]. Four studies **[**16, 41–43] used the arm to estimate *P*_mcf_. For arm occlusion, a rapid cuff inflator (inflates in 0.3 s) [16, 43] or a pneumatic tourniquet (inflates in 1.4 s) [41, 42] was inflated around the upper arm to 50 mmHg above systolic blood pressure. Arterial and venous pressures were measured via a radial artery catheter and a peripheral venous cannula in the forearm. When these two pressures equalize, *P*_mcf_ arm values are achieved. An initial study determined that a 25–30 s stop-flow time was adequate to achieve this equilibration [16]. Following this, in two studies *P*_mcf_ arm was measured as the average radial arterial pressure at 30 s after stop-flow [16, 43]. One study found the smallest difference between venous and arterial pressure after 60 s of stop-flow [41]. This discrepancy could be explained by different inflation time, i.e., induction of stop-flow.

### Clinical applicability

The average baseline *P*_mcf_ arm values found in the included studies range from 16 to 24 mmHg (Table 2). *P*_mcf_ arm can be performed in spontaneously breathing subjects and requires only one measure. In two studies, *P*_mcf_ arm was assessed as a predictor of fluid loading responsiveness (FLR) [16, 43]. One study showed that a low *P*_mcf_ arm (< 22 mmHg) predicts FLR with 71% sensitivity and 88% specificity, where responders were defined when CO increased > 10% after 500 mL colloid administration [43]. Another study showed changes in circulating volume (500 mL colloid) are tracked well by changes in *P*_mcf_ arm [16]. Finally, one study indicated a minimum of 4 mL/kg fluid challenge was needed to define FLR [42].

### Precision and accuracy

Repeated measurements of *P*_mcf_ arm showed no significant differences [41]. The coefficient of variation for a single measurement was 5%, which reduced to 3% after four measurements. Bland–Altman analysis showed a bias of − 0.1 ± 1.68 mmHg for the first two measurements. The least significant change [44] for a single measurement was 14% (i.e., ± 3 mmHg for a *P*_mcf_ arm of 22 mmHg). One study observed a negligible bias of two *P*_mcf_ arm determinations at baseline position and after fluid expansion [16]. Two studies [16, 30] found no significant differences in *P*_mcf_ arm to *P*_mcf_ hold measures.

### Limitations

Theoretically, a limitation of the technique is the influence of an auto regulatory hypoxia-induced response causing arterial vasodilation. The time of measuring *P*_mcf_ after arm occlusion should be enough for arterial and venous pressures to equilibrate, but before hypoxia-induced vasodilation causes an underestimation of *P*_mcf_ [45]. One study observed plateau pressures after 20–30 s and saw a further decrement after 35–40 s which indicates hypoxia-induced vasodilation [16]. Potentially, arm occlusion causes a small accumulation of blood volume because the venous outflow stops before the arterial inflow stops [16]. Though, this potential overestimation is negligible since the inflow is small compared to the total distal arm volume as long as cuff inflation is rapid. To note, *P*_mcf_ arm is only reliable when a stable plateau pressure is achieved [2].

In contrast to *P*_mcf_ hold, *P*_mcf_ arm measures can be made in non-sedated patients with cardiac arrhythmias. However, the possible influence of the rapid cuff inflator on reflex mechanisms needs to be studied. In septic patients, central and peripheral vasomotor tone might be altered differently [46]. Shortly after cardiac surgery differences between aortic and radial pressure can occur [47], still, the original validation studies were on postoperative cardiac surgery patients.

## *P*_mcf_ analogue

### Technique description

Based on a Guytonian model of the systemic circulation (CO = *V*_R_ = (*P*_mcf_ − CVP)/RVR), an analogue of *P*_mcf_ can be derived using a mathematical model: *P*_mcf_ analogue = axCVP + bxMAP + cxCO [5, 20, 48, 49]. In this formula, *a* and *b* are dimensionless constants (*a* + *b* = 1). Assuming a veno-arterial compliance ratio of 24:1, *a* = 0.96 and *b* = 0.04; c resembles arteriovenous resistance and is based on a formula including age, height and weight [5, 48–50].

### Clinical applicability

The average baseline *P*_mcf_ analogue values found in the included studies range from 14 to 18 mmHg (Table 2). One study compared fluid replacement based on target *P*_mcf_ analogue compared to conventional treatment in continuous veno-venous hemodiafiltration [49]. Fluid replacement based on target *P*_mcf_ analogue led to significantly less fluid administration with stable cardiovascular variables (CVP, MAP, CO) and no complications. So, *P*_mcf_ analogue measurement adequately follows intravascular volume status in patients. *P*_mcf_ analogue measurements are automatic making it an attractive alternative to *P*_mcf_ hold and *P*_mcf_ arm.

More recently, the *P*_mcf_ analogue dynamics, measured with the Navigator™ device (Applied Physiology, Pty Ltd, Australia), were observed [20, 48, 51]. Patients were defined as responders with an increase in stroke volume or CO > 10% after 250 mL fluid administration. *P*_mcf_ analogue increased after fluid administration; however, baseline *P*_mcf_ analogue did not differ between responders and non-responders [20, 45, 48] (Table 2). This is contrary to results of another study [43] using *P*_mcf_ arm, possibly due to different fluid volume (250 vs. 500 mL) [42]. Although the driving pressure for *V*_R_ (*P*_mcf_ CVP) was different between responders and non-responders, it showed low sensitivity (79%) and specificity (56%) to predict FLR [20, 48].

### Precision and accuracy

Precision has not been assessed for *P*_mcf_ analogue (Table 4). Comparing measurement techniques revealed a lower *P*_mcf_ analogue value compared to *P*_mcf_ hold [16]. However, a significant regression of *P*_mcf_ analogue and *P*_mcf_ hold was observed enabling to adjust the *P*_mcf_ analogue value using calibration factor [5].

**Table 4:** Comparison of bedside *P*_mcf_ measurement techniques

	*P*_mcf_ hold	*P*_mcf_ arm	*P*_mcf_ analogue
	CO = (*P*_mcf_ CVP)/RVR	*P*_a_ = *P*_v_	*P*_mcf_ = axCVP + bxMAP + cxCO
Applicability to a broad patient population	−	±	±
	Restricted to fully sedated and mechanically ventilated patients	In theory applicable in all patients (sedated or awake) with an radial artery catheter	In theory applicable in all patients (sedated or awake)
	Restricted to patients without a contraindication for inspiratory holds (such as COPD with bullae)		Continuous and accurate CO, MAP and CVP measurements needed
	Continuous and accurate CO and CVP measurements needed		Not suitable in cardiac arrhythmia
	Not suitable in cardiac arrythmia		
Accuracy	+	+	−
	Values interchangeable with *P*_mcf_ arm	Values interchangeable with *P*_mcf_ hold	Values significantly lower than derived with *P*_mcf_ hold
	When sedated baroreflex probably of little influence	Dependent on time of measurement: > *P*_a_ and *P*_v_ equilibration. < hypoxia-induced vasodilatation	*P*_mcf_ analogue can be transformed to *P*_mcf_ hold values (constant error)
	Mechanical ventilation may overestimate *P*_mcf_ value	Possible influence rapid cuff inflator on reflex mechanism altering *P*_mcf_ value in non-sedated patients. This is not studied	Mathematical coupling and the equation is based on assumptions that may not be generalizable to all patient populations in ICU
Precision	?	+	?
	Not studied	No significant differences during repeated measurements. LSC for a single measurement is 14%	Not studied
Outcome operator independent	−	±	+
	Inspiratory holds	Timing of measurement	CVP transducer position and CO measurement technique
	CVP transducer position and CO measurement technique		
	Extrapolation of curve		
Responding time	−	+	+
	> 4 min	30–60 s	Fast, no exact times mentioned
Costs	−	+	+
	Theoretically no extra devices needed than standard present in ICU	Rapid Cuff Inflator (Hokanson E20, Bellevue, Washington, USA) = 3000 euro	Navigator™ (Applied Physiology, Pty Ltd, Sydney, Australia)
			Price unknown
Risk of complications	+	±	−
	No complications reported in published studies. In theory:	No complications reported in published studies. In theory:	No complications reported in published studies. In theory:
	Barotrauma from inspiratory holds	In sedated patients attention should be paid deflating the rapid cuff before hypoxemia-induced damage can occur	Complications associated with central venous catheters and CO measurement
	Severe hemodynamic instability induced by inspiratory holds	In awake patients local pain could be caused by inflating the rapid cuff inflator	
	Complications associated with central venous catheters and CO measurement		

*CO* cardiac output, *CVP* central venous pressure, *RVR* resistance to venous return, *MAP* mean arterial pressure, *P*_a_ arterial pressure, *P*_v_ venous pressure (the latter two measured in the arm)

### Limitations

The mathematical model is based on CVP, MAP and CO measurements. As CVP values vary during ventilation, usually end-expiratory CVP-recordings can be used. Furthermore, CVP values depend on the position of the transducer. Accurate CO values are needed for this method. The limitation of *P*_mcf_ analogue is that the algorithm is based on a mathematical model with mathematical coupling between CO and *P*_mcf_ and fixed Csys and resistance parameters [5], therefore presumably not applicable for all patient populations or clinical conditions. We are unable to assess the availability of the Navigator™ for routine care.

## Discussion

We found three bedside techniques to measure *P*_mcf_: *P*_mcf_ hold, *P*_mcf_ arm and *P*_mcf_ analogue. They were used to follow volumetric state and to study drug-induced hemodynamic changes in patients.

The interpretation of *V*_R_ curves and *P*_mcf_ in clinical practice is subject to debate [52–59]. The values found in heart-beating ICU patients are higher (14–33 mmHg) than in deceased ICU patients (12.8 ± 5.6 mmHg, mean ± sd), probably because of alteration of vasomotor tone after dying [53]. Furthermore, ICU patients often receive vasopressors which increase *P*_mcf_ and the study populations differed making it not one-to-one comparable. It is also speculated that the pressure described by Guyton is not measurable in heart-beating patients and the extrapolated pressure of the curve represents a different physiological parameter. Nevertheless, in two studies *P*_mcf_ arm was interchangeable with *P*_mcf_ hold [16–30]. Furthermore, although *P*_mcf_ values may differ, the CVP values do as well, which may account for a similar driving pressure for *V*_R_. The reviewed studies illustrate the possible clinical benefits of using the bedside derived *P*_mcf_ values.

This review is limited since we were unable to pool the data because of the variety in used conditions and interventions. The 16 included studies were performed by only a few research groups with a limited amount of included patients. In most of the studies, each patient served as their own control since it is not clear what would be an appropriate outside control group.

Still, all studies testing the accuracy of *P*_mcf_ to follow intravascular changes and pharmacodynamics found significant results. Therefore, it is unlikely that a larger number of patients will show different outcomes. It is possible only positive studies were published, indicating publication bias. *P*_mcf_ values differ between the studies and have a wide range within studies (Table 2). Normal values for different patient populations need to be defined before *P*_mcf_ can be implemented into standard (ICU) care. The increase in *P*_mcf_ values after fluid administration depends on vascular redistribution, vasomotor tone and fluid loss into the interstitial space. Studies focusing on clinical decision-making based on *P*_mcf_, driving pressure for *V*_R_, *V*_s_ or Csys have not yet been performed. Study designs need to be created to see if using these measures improves outcomes. Also, no precision studies examining *P*_mcf_ hold or *P*_mcf_ analogue exist yet.

## Conclusions

Presently, three bedside *P*_mcf_ measurement techniques are available. All require invasive hemodynamic monitoring. Though *P*_mcf_ measures allow for more direct assessment of circulating blood volume, *V*_R_ and Csys, studies are needed to determine cutoff values to allow *P*_mcf_ to trigger therapeutic interventions and to determine its value in clinical practice.

## Abbreviations

CO: cardiac output; Csys: vascular compliance, CVP: central venous pressure; FiO_2_: fractional oxygen concentration; FLR: fluid loading responsiveness; ICU: intensive care unit; MAP: mean arterial pressure; RVR: resistance for venous return.

## List of symbols

*P*_cc_: critical closing pressure; *P*_mcf_: mean circulatory filling pressure; *P*_ra_: right atrial pressure; *V*_R_: venous return; *V*_s_: stressed volume; *V*_u_: unstressed volume.

## Additional file

**Additional file 1.** **I: Search in EMBASE, MEDLINE and Cochrane Library**: Description of the used search terms per database. **II: Quality assessment according to a modified version of the Newcastle–Ottawa scale for cohort studies**: Including representativeness, ascertainment, demonstration, comparability and outcome. **III: PRISMA Flowchart**: Description of results of systematic literature search, reasons for excluding studies and the amount of included studies. **IV: Expanded baseline characteristics for included studies**: Authors, described *P*_mcf_ measurement method, patient population, exclusion criteria, age and sex of included patients, type of cardiac output measurement, used vasopressors, sedation and anesthesia techniques and timeframes of *P*_mcf_ measurements. **V: PRISMA 2009 Checklist**: an evidence-based minimum set of items for reporting in systematic reviews and meta-analysis.
