# Supplementary material for: Estimating mean circulatory filling pressure in clinical practice: a systematic review comparing three bedside methods in the critically ill
Source: Ann Intensive Care. 2018 Jun 20;8:73. doi: 10.1186/s13613-018-0418-2 (PMC6010367; doi:10.1186/s13613-018-0418-2)
Supplement: Supplementary file 1 — Additional file 1. I: Search in EMBASE, MEDLINE and Cochrane Library: Description of the used search terms per database. II: Quality assessment according to a modified version of the Newcastle–Ottawa scale for cohort studies: Including representativeness, ascertainment, demonstration, comparability and outcome. III: PRISMA Flowchart: Description of results of systematic literature search, reasons for excluding studies and the amount of included studies. IV: Expanded baseline characteristics for included studies: Authors, described Pmcf measurement method, patient population, exclusion criteria, age and sex of included patients, type of cardiac output measurement, used vasopressors, sedation and anesthesia techniques and timeframes of Pmcf measurements. V: PRISMA 2009 Checklist: an evidence-based minimum set of items for reporting in systematic reviews and meta-analysis. [file 13613_2018_418_MOESM1_ESM.doc]

**Additional files**

**Title:** Estimating mean circulatory filling pressure in clinical practice: A systematic review comparing three bedside methods

**Authors:** Marije Wijnberge MD^1,2,3^, Daniko P. Sindhunata BSc^1^ , Michael R. Pinsky MD^5^, Alexander P. Vlaar MD PhD MBA^2,3^, Else Ouweneel MA PhD Msc^1^, Jos R. Jansen MSc PhD^4^, Denise P. Veelo MD PhD^1^, Bart F. Geerts MD PhD MSc MBA^1^

**Affiliations:**

1. Academic Medical Center, Department of Anesthesiology, Amsterdam, The Netherlands
2. Academic Medical Center, Department of Intensive Care, Amsterdam, The Netherlands
3. Academic Medical Center, Laboratory of Experimental Intensive Care and Anesthesiology, Amsterdam, The Netherlands
4. Leiden University Medical Center, Department of Intensive Care Medicine, Leiden, The Netherlands
5. University of Pittsburgh Medical Center, Department of Critical Care Medicine, Pittsburgh, USA

**Corresponding Author:**

Professor Michael R. Pinsky MD, CM, Dr hc

Department of Critical Care Medicine

1215.4 Lillian S. Kaufmann Bldg, 3471 Fifth Avenue, Pittsburgh, PA 15213

T (412) 647-7766

E [pinsky@pitt.edu](mailto:pinsky@pitt.edu)

**Legend Additional files**

**I: Search in EMBASE, MEDLINE and Cochrane Library**: Description of the used search terms per database.

**II: Quality assessment according to a modified version of the Newcastle-Ottowa scale for cohort studies:** Including representativeness, ascertainment, demonstration, comparability and outcome.

**III: PRISMA Flowchart**: Description of results of systematic literature search, reasons for excluding studies and the amount of included studies.

**IV: Expanded baseline characteristics for included studies**: Authors, described Pmcf measurement method, patient population, exclusion criteria, age and sex of included patients, type of cardiac output measurement, used vasopressors, sedation and anesthesia techniques and timeframes of Pmcf measurements.

**V: PRISMA 2009 Checklist**: an evidence-based minimum set of items for reporting in systematic reviews and meta-analysis.

**Additional file I: Search in EMBASE, MEDLINE and Cochrane Library**

The specific search terms were as follows:

**EMBASE (Ovid)** 133 hits

Database(s): **Embase Classic+Embase**1947 to 2017 May 18

Search Strategy:

| **#** | **Searches** | **Results** |
| --- | --- | --- |
| 1 | (mean systemic filling pressure* or MSFP or PMSF or mean circulatory filling pressure* or static filling pressure* or mean static filling pressure* or mean systemic pressure*).ti,ab,kw. | 2384 |
| 2 | exp intensive care/ or exp intensive care unit/ or exp intensive care nursing/ or perioperative period/ or surgery/ or perioperative period/ or surgery/ or perioperative nursing/ or peroperative care/ or exp intraoperative period/ or exp intraoperative monitoring/ or (surgery or surgical or IC or ICU* or intensive care or critical care or perioperat* or peri-operat* or intraoperat* or intra-operat* or intravascular).ti,ab,kw. | 2975277 |
| 3 | 1 and 2 | 133 |

**MEDLINE (Pubmed)** 193 hits

(mean systemic filling pressure*[tiab] OR MSFP[tiab] OR PMSF[tiab] OR mean circulatory filling pressure*[tiab] OR static filling pressure*[tiab] OR mean systemic pressure*[tiab]) AND ("Critical Care"[Mesh] OR "Intensive Care Units"[Mesh] OR "Critical Care Nursing"[Mesh] OR "Perioperative Period"[Mesh] OR "Perioperative Care"[Mesh] OR "Perioperative Nursing"[Mesh] OR "Intraoperative Care"[Mesh] OR "Intraoperative Period"[Mesh] OR "Monitoring, Intraoperative"[Mesh] OR "Surgical Procedures, Operative"[Mesh] OR "General Surgery"[Mesh] OR "surgery" [Subheading] OR surgery OR surgical OR IC OR ICU* OR intensive care OR critical care OR perioperat* OR intraoperat* OR peri-operat* OR intra-operat*OR intravascular)

**Cochrane Library** 53 hits

ID Search Hits

#1 mean systemic filling pressure* or MSFP or PMSF or mean circulatory filling pressure* or static filling pressure* or mean static filling pressure*: ti, ab, kw (Word variations have been searched) 136

#2 MeSH descriptor: [Critical Care] explode all trees 2131

#3 MeSH descriptor: [Intensive Care Units] explode all trees 3301

#4 MeSH descriptor: [Critical Care Nursing] explode all trees 22

#5 MeSH descriptor: [Perioperative Period] explode all trees 7342

#6 MeSH descriptor: [Perioperative Care] explode all trees 11730

#7 MeSH descriptor: [Perioperative Nursing] explode all trees 130

#8 MeSH descriptor: [Intraoperative Care] explode all trees 1476

#9 MeSH descriptor: [Intraoperative Period] explode all trees 2018

#10 MeSH descriptor: [Monitoring, Intraoperative] explode all trees 1514

#11 MeSH descriptor: [Surgical Procedures, Operative] explode all trees 116933

#12 MeSH descriptor: [General Surgery] explode all trees 365

#13 surgery or surgical or IC or ICU* or intensive care or critical care or perioperat* or peri-operat* or intraoperat* or intra-operat* or intravascular: ti, ab, kw (Word variations have been searched) 157285

#14 #2 or #3 or #4 or #5 or #6 or #7 or #8 or #9 or #10 or #11 or #12 or #13 213997

#15 #1 and #14 53

**Additional file II: Quality assessment according to a modified version of the Newcastle-Ottowa scale**

| **Study** | **Method** | **Representativeness** | **Ascertainment** | **Demonstration** | **Comparability** | **Outcome** | **Total stars** |
| --- | --- | --- | --- | --- | --- | --- | --- |
| **Maas (1)**  **2009** | Pmcf-hold | ***** | ***** | ***** |  | ***** | **4** |
| **Keller (2)**  **2011** | Pmcf-hold | ***** | ***** | ***** |  | ***** | **4** |
| **Maas (3)**  **2012** | Pmcf-hold | ***** | ***** | ***** |  | ***** | **4** |
| **Persichini (4)**  **2012** | Pmcf-hold | ***** | ***** | ***** |  | ***** | **4** |
| **Maas (5)**  **2013** | Pmcf-hold | ***** | ***** | ***** |  | ***** | **4** |
| **Guerin (6)**  **2015** | Pmcf-hold | ***** | ***** | ***** |  | ***** | **4** |
| **De Wit (7)**  **2016** | Pmcf-hold | ***** | ***** | ***** |  | ***** | **4** |
| **Helmerhorst (8)**  **2017** | Pmcf-hold | ***** | ***** | ***** |  | ***** | **4** |
| **Geerts (9)**  **2011** | Pmcf-arm | ***** | ***** | ***** |  | ***** | **4** |
| **Aya (10)**  **2014** | Pmcf-arm | ***** | ***** | ***** |  | ***** | **4** |
| **Aya (11)**  **2017** | Pmcf-arm | ***** | ***** | ***** |  | ***** | **4** |
| **Parkin (12)**  **1994** | Pmcf-analogue |  | ***** | ***** |  | ***** | **3** |
| **Cecconi (13)**  **2013** | Pmcf-analogue | ***** | ***** | ***** |  | ***** | **4** |
| **Gupta (14)**  **2015** | Pmcf-analogue | ***** | ***** | ***** |  | ***** | **4** |
| **Aya (15)**  **2016** | Pmcf-analogue | ***** | ***** | ***** |  | ***** | **4** |
| **Maas (16)**  **2012** | Pmcf-hold  Pmcf-arm  Pmcf-analogue | ***** | ***** | ***** | ***** | ***** | **5** |
| **Maas (17)**  **2012** | Pmcf-hold  Pmcf-arm | ***** | ***** | ***** | ***** | ***** | **5** |

**Additional file II:** Quality assessment based on a modified version of the Newcastle-Ottowa scale for cohort studies (18).

**Additional file III: PRISMA Flowchart**

**Screening**

**Included**

**Eligibility**

**Identification**

Pubmed
(n = 193)

Cochrane
(n = 53)

Embase
(n = 133)

Citation tracking
(n = 2)

Records after duplicates removed
(n = 370)

Records screened
(n = 370)

Records excluded
(n = 300)

Full-text articles excluded:
Conference abstract (n = 13)

Letters to the editor (n = 7)

Opinion pieces / reviews (n = 20)

VF used as method (n = 2)

Deceased patients (n = 1)

Full text not available in English (n = 3)

Erratum (n = 1)

Studies performed in children (n=2)

Alternative outcomes (n=2)

Non-ICU (n = 2)

Full-text articles assessed for eligibility
(n = 70)

Studies included
(n = 17)

Pmcf-analogue
(n = 4)

Comparison
(n = 2)

Pmcf-hold

(n = 8)

Pmcf-arm
(n = 3)

**Additional file III:** PRISMA flowchart (19).

**Additional file IV: Baseline characteristics for included studies**

| **Study** | **Method** | **N** | **Patient population**  **(all adult ICU patients)** | **Exclusion criteria** | **Age** | **Male** | **Cardiac output measurement** | **Vasopressor use** | **Sedation/**  **Anaesthesia** | **Timeframe Pmcf measurement** |
| --- | --- | --- | --- | --- | --- | --- | --- | --- | --- | --- |
| **Maas (1)**  **2009** | Pmcf-hold | 12 | Postoperative cardiac surgery  10 CABG  2 AVR | CHF NYHA IV  Aortic aneurysm  Extensive PAOD  Valvular insufficiency  Arrhythmia  Artificial pacing  Cardiac assist device | 64 (10) | 10 (83%) | Beat-to-beat CO  Modelflow pulse contour analysis  Calibrated with thermodilution | 9 patients  Dobu  NE  NPN  Enox | All patients  Propofol  Sufentanil | Not described |
| **Keller (2)**  **2011** | Pmcf-hold | 9 | Postoperative cardiac surgery  3 CABG  6 AVR | LVEF<45%  Aortic aneurysms  PAOD  Valvular insufficiency Arrhythmia  Artificial pacing  Cardiac assist device | Median 61  IQR 55-75 | 4 (44%) | Beat-to-beat CO  Pulse contour analysis (PiCCO)  Calibrated with thermodilution | None | All patients  Propofol  Sufentanil | Not described |
| **Maas (3)**  **2012** | Pmcf-hold | 10 | Postoperative cardiac surgery  2 AVR  1 MVP +TVP  7 CABG | CHF NYHA IV  Aortic aneurysm  Extensive PAOD  Valvular insufficiency  Arrhythmia  IABP | 64 (11) | 9 (90%) | Beat-to-beat CO  Modelflow pulse contour analysis  Calibrated with thermodilution(*) | 8 patients  NE  NPN  Dobu | All patients  Propofol  Sufentanil | Within 1 hour after ICU admission |
| **Persichini (4)**  **2012** | Pmcf-hold | 16 | Septic shock | Pregnancy  PLR contraindicated | 67 (16) | 8 (50%) | Beat-to-beat CI  pulse contour analysis (PiCCO2)  Calibrated with thermodilution. | All patients  NE | All patients received sedation (not specified) | Not described |
| **Maas (5)**  **2013** | Pmcf-hold | 16 | Postoperative cardiac surgery  1 MVP  15 CABG | Previous myocardial infarction  LVEF<45%  Aortic insufficiency  Aortic aneurysm  Extensive PAOD | 64 (11) | Not described | Beat-to-beat CO  Modelflow pulse contour analysis  Calibrated with lithium indicator dilution method (LiDCO) | All patients  NE  1 patient Dobu | All patients  Propofol  Sufentanil | Within 1 hour after ICU admission |
| **Guerin (6)**  **2015** | Pmcf-hold | 30 | Shock aetiology  9 Septic Shock  4 Cardiogenic Shock  2 Hypovolemic Shock | PLR contraindicated | 65 (12) | 21 (70%) | Beat-to-beat CI  pulse contour analysis (PiCCO2)  Calibrated with thermodilution. | 23 patients  NE  3 patients Dobu | 29 patients  Propofol  13 patients  Remifentanil | Not described |
| **De Wit (7)**  **2016** | Pmcf-hold | 17 | Postsurgical  16 oesophageal resection  1 pancreaticoduodenectomy | Aberrant cardiovascular anatomy  Significant valvular regurgitation  Severe arrhythmias | 62 (9) | 14 (82%) | Beat-to-beat CO  Modelflow pulse contour analysis  Calibrated with thermodilution (*) | 1 patient  NE | All patients  Propofol | Not described |
| **Helmerhorst (8)**  **2017** | Pmcf-hold | 22 | Postoperative cardiac surgery  22 CABG | CHF  Severe arrhythmias  Intracardiac shunts  Extensive PAOD  Pulmonary disease  Aortic aneurysm Significant valvular disease | 63 (59-66) | 17 (85%) | Beat-to-beat CO obtained by Modelflow pulse contour analysis. Hemodynamics also monitored by LiDCO*plus* | 2 patients  NE | All patients  Propofol  Sufentanil | 1 hour after ICU admission |
| **Geerts (9)**  **2011** | Pmcf-arm | 24 | Postoperative cardiac surgery  17 CABG  7 CABG plus valve repair | Aortic aneurysm Extensive PAOD Arrhythmias Postoperative valvular insufficiency  Artificial pacing  Cardiac assist device | 64 (10) | 19 (79%) | CO not required for Pmcf measurement | 16 NE  9 Dobu  1 NPN | All patients  Propofol  Sufentanil | Within 2 hour after ICU admission |
| **Aya (10)**  **2014** | Pmcf-arm | 20 | Postoperative cardiac surgery  13 CABG  4 AVR  4 MVR | Extensive PAOD  Postoperative valve regurgitation Tachyarrhythmia  IABP  Pregnancy  Body weight below 50kg | 63 (11) | 17 (85%) | CO not required for Pmcf measurement | 13 NE  4 Dopa  3 Milrinone | 16 propofol  11 morphine  2 alfentanyl | Initial period at ICU (not further defined) |
| **Aya (11)**  **2017** | Pmcf-arm | 80 | Postoperative cardiac surgery  36 CABG  27 AVR+CABG  12 MVR+CABG  5 Other | Extensive PAOD  Postoperative valve regurgitation Tachyarrhythmia  IABP  Pregnancy  Body weight below 50kg Active bleeding  Sepsis | 70  Range  52-80 | 62 (78%) | CO not required for Pmcf measurement | 43 patients  Dopa or NE | 26 patients  Propofol or Morphine | Initial period at ICU (not further defined) |
| **Parkin (12)**  **1994** | Pmcf-analogue | 10 | Multi-organ failing patients receiving CVVH for acute renal failure | Not described | 65  Range  24-77 | 7 (70%) | Thermodilution CO measured **each hour** | All patients inotropic or vasoactive medication. (not specified) | Not described | Not described |
| **Cecconi (13)**  **2013** | Pmcf-analogue | 39 | Postoperative fluid challenge  22 Cardiac surgery  8 Shock  6 Non cardiac surgery  3 Other | Aortic regurgitation Tachyarrhythmia IABP Pregnancy Body weight below 50 kg | 68 (12) | 26 (67%) | Beat-to-beat CO  Pulse contour analysis with LiDCO plus. Calibrated with lithium-dilution | 2 NE  5 Dopa  4 Milrinone | Not described | Not described |
| **Gupta (14)**  **2015** | Pmcf-analogue | 61 | Postoperative cardiac surgery  40 CABG  8 CABG + valve replacement  8 Valve replacement  5 Bentall’s procedure  7 DDD pacing | Not described  To note: patients with arrhythmia, paced rhythms and spontaneous breathing efforts were included | 63 (11) | 46 (75%) | PAC thermodilution (not continuous) | 27 NE  6 Dobu  10Milnirone  9 NPN  6 Glyceryl trinitrate | All patients  Propofol  Fentanyl  or Morphine | Within 6 hours after ICU admission |
| **Aya (15)**  **2016** | Pmcf-analogue | 26 | Postoperative fluid challenge  7 Cardiac surgery  19 Noncardiac surgery | Extensive PAOD  Postoperative valve regurgitation Tachyarrhythmia IABP  Pregnancy  Body weight below 50kg Active bleeding  Sepsis | 68  Range  53-80 | 16 (62%) | Beat-to-beat CO  LiDCOplus pulse power analysis Calibrated with lithium dilution | 9 NA  1 Dopa  2 Dopexamine  1 Dobu  3 Milrinone  1Adrenaline | 14 patients  Propofol | Initial period at ICU (not further defined) |
| **Maas (16)**  **2012** | Pmcf-hold  Pmcf-arm  Pmcf-analogue | 11  11  11 | Postoperative cardiac surgery  9 CABG  2 AVR | LVEF<40%  Aortic aneurysm  Extensive PAOD Postoperative arrhythmia Postoperative valvular insufficiency  Artificial pacing  Cardiac assist device | 64  Range  50-80 | 9 (82%) | Beat-to-beat CO  Modelflow pulse contour analysis Calibrated with thermodilution. | 4 Dobu  1 Enox  5 NE  1 NPN | All patients  Propofol  Sufentanil | Within 2 hours after ICU admission |
| **Maas (17)**  **2012** | Pmcf-arm  Pmcf-hold | 15  12 | Postoperative cardiac surgery  9 CABG  5 Valve  1 CABG + valve | NYHA IV  Aortic aneurysm Extensive PAOD  Arrhythmias | 64 (11) | Not described | Beat-to-beat CO  Modelflow pulse contour analysis  Calibrated with thermodilution (*) | 8 Dobu  1 Enox  7 NE  1 Epinephrine  1 NPN | Propofol  Sufentanil | Within 1 hour after ICU admission |

**Additional file IV**: Baseline characteristics for included studies. CHF= congestive heart failure. CABG= coronary artery bypass, MVR= mitral valve replacement, MPV= mitral valve prolapse, AVR= aortic valve replacement, TVP= tricuspid valve prolapse, NYHA=New York Heart Association scoring system, PAOD= peripheral arterial occlusive disease, LVEF= left ventricular ejection fraction. CO= cardiac output, CVVH=continuous veno-venous hemodiaflitration, IABP= intra-aortic balloon pump, Dobu= dobutamine, NE= norepinephrine, NPN= nitroprusside sodium, Dopa= dopamine, Enox= enoximone, GI= gastrointestinal. (*) Calibration techniques not available in the articles, authors contacted. Age is presented as mean with standard deviation (SD) or median with range or interquartile range (IQR). Number of males per study is presented as counts with percentage.

**Additional file V: PRISMA 2009 Checklist**


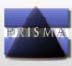
**PRISMA 2009 Checklist**

| **Section/topic** | **#** | **Checklist item** | **Reported on page #** | |
| --- | --- | --- | --- | --- |
| **TITLE** | | | |  |
| Title | 1 | Identify the report as a systematic review, meta-analysis, or both. | | # 1 |
| **ABSTRACT** | | | |  |
| Structured summary | 2 | Provide a structured summary including, as applicable: background; objectives; data sources; study eligibility criteria, participants, and interventions; study appraisal and synthesis methods; results; limitations; conclusions and implications of key findings; systematic review registration number. | | # 2 |
| **INTRODUCTION** | | | |  |
| Rationale | 3 | Describe the rationale for the review in the context of what is already known. | | # 3 |
| Objectives | 4 | Provide an explicit statement of questions being addressed with reference to participants, interventions, comparisons, outcomes, and study design (PICOS). | | # 3-4 |
| **METHODS** | | | |  |
| Protocol and registration | 5 | Indicate if a review protocol exists, if and where it can be accessed (e.g., Web address), and, if available, provide registration information including registration number. | | - |
| Eligibility criteria | 6 | Specify study characteristics (e.g., PICOS, length of follow-up) and report characteristics (e.g., years considered, language, publication status) used as criteria for eligibility, giving rationale. | | # 4 |
| Information sources | 7 | Describe all information sources (e.g., databases with dates of coverage, contact with study authors to identify additional studies) in the search and date last searched. | | # 4 and additional file I |
| Search | 8 | Present full electronic search strategy for at least one database, including any limits used, such that it could be repeated. | | Additional file I |
| Study selection | 9 | State the process for selecting studies (i.e., screening, eligibility, included in systematic review, and, if applicable, included in the meta-analysis). | | # 4 and additional file III |
| Data collection process | 10 | Describe method of data extraction from reports (e.g., piloted forms, independently, in duplicate) and any processes for obtaining and confirming data from investigators. | | # 4 |
| Data items | 11 | List and define all variables for which data were sought (e.g., PICOS, funding sources) and any assumptions and simplifications made. | | #4, tables and additional file IV |
| Risk of bias in individual studies | 12 | Describe methods used for assessing risk of bias of individual studies (including specification of whether this was done at the study or outcome level), and how this information is to be used in any data synthesis. | | # 4 |
| Summary measures | 13 | State the principal summary measures (e.g., risk ratio, difference in means). | | # 4 |
| Synthesis of results | 14 | Describe the methods of handling data and combining results of studies, if done, including measures of consistency (e.g., I^2^) for each meta-analysis. | | Not applicable |
| **Section/topic** | **#** | **Checklist item** | **Reported on page #** | |
| Risk of bias across studies | 15 | Specify any assessment of risk of bias that may affect the cumulative evidence (e.g., publication bias, selective reporting within studies). | #4, additional file II and # 11 | |
| Additional analyses | 16 | Describe methods of additional analyses (e.g., sensitivity or subgroup analyses, meta-regression), if done, indicating which were pre-specified. | Not applicable | |
| **RESULTS** | | |  | |
| Study selection | 17 | Give numbers of studies screened, assessed for eligibility, and included in the review, with reasons for exclusions at each stage, ideally with a flow diagram. | #4 and additional file III | |
| Study characteristics | 18 | For each study, present characteristics for which data were extracted (e.g., study size, PICOS, follow-up period) and provide the citations. | #4, #5, tables and additional file IV | |
| Risk of bias within studies | 19 | Present data on risk of bias of each study and, if available, any outcome level assessment (see item 12). | Additional file II | |
| Results of individual studies | 20 | For all outcomes considered (benefits or harms), present, for each study: (a) simple summary data for each intervention group (b) effect estimates and confidence intervals, ideally with a forest plot. | Not applicable | |
| Synthesis of results | 21 | Present results of each meta-analysis done, including confidence intervals and measures of consistency. | Not applicable | |
| Risk of bias across studies | 22 | Present results of any assessment of risk of bias across studies (see Item 15). | #11 | |
| Additional analysis | 23 | Give results of additional analyses, if done (e.g., sensitivity or subgroup analyses, meta-regression [see Item 16]). | Not applicable | |
| **DISCUSSION** | | |  | |
| Summary of evidence | 24 | Summarize the main findings including the strength of evidence for each main outcome; consider their relevance to key groups (e.g., healthcare providers, users, and policy makers). | #11 | |
| Limitations | 25 | Discuss limitations at study and outcome level (e.g., risk of bias), and at review-level (e.g., incomplete retrieval of identified research, reporting bias). | #7-11 | |
| Conclusions | 26 | Provide a general interpretation of the results in the context of other evidence, and implications for future research. | #10-11 | |
| **FUNDING** | | |  | |
| Funding | 27 | Describe sources of funding for the systematic review and other support (e.g., supply of data); role of funders for the systematic review. | #12-13 | |

**Additional file V: PRIMSA 2009 Checklist (20)**

**References**

1. Maas JJ, Geerts BF, van den Berg PC, Pinsky MR, Jansen JR. Assessment of venous return curve and mean systemic filling pressure in postoperative cardiac surgery patients. Crit Care Med. 2009;37:912-8.

2. Keller G, Desebbe O, Benard M, Bouchet JB, Lehot JJ Bedside assessment of passive leg raising effects on venous return. J Clin Monit Comput. 2011;25:257-63.

3. Maas JJ, de Wilde RB, Aarts LP, Pinsky MR, Jansen JR. Determination of vascular waterfall phenomenon by bedside measurement of mean systemic filling pressure and critical closing pressure in the intensive care unit. Anesth Analg. 2012;114:803-10.

4. Persichini R, Silva S, Teboul JL, Jozwiak M, Chemla D, Richard C, et al. Effects of norepinephrine on mean systemic pressure and venous return in human septic shock. Crit Care Med. 2012;40:3146-53.

5. Maas JJ, Pinsky MR, de Wilde RB, de Jonge E, Jansen JR. Cardiac output response to norepinephrine in postoperative cardiac surgery patients: interpretation with venous return and cardiac function curves. Crit Care Med. 2013;41:143-50.

6. Guerin L, Teboul JL, Persichini R, Dres, M, Richard C, Monet X. Effects of passive leg raising and volume expansion on mean systemic pressure and venous return in shock in humans. Critical Care. 2015; 19:411

7.de Wit F, van Vliet AL, de Wilde RB, Jansen JR, Vuyk J, Aarts LP, et al. The effect of propofol on haemodynamics: cardiac output, venous return, mean systemic filling pressure, and vascular resistances. Br J Anaesth. 2016;116:784-9.

8. Helmerhorst HJ, de Wilde RB, Lee DH, Palmen M, Jansen JR, van Westerloo DJ, et al. Hemodynamic effects of short-term hyperoxia after coronary artery bypass grafting. Ann Intensive Care. 2017;7:20.

9. Geerts BF, Maas J, de Wilde RB, Aarts LP, Jansen JR. Arm occlusion pressure is a useful predictor of an increase in cardiac output after fluid loading following cardiac surgery. Eur J Anaesthesiol. 2011;28:802-6.

10. Aya HD, Rhodes A, Fletcher N, Grounds RM, Cecconi M. Transient stop-flow arm arterial-venous equilibrium pressure measurement: determination of precision of the technique. J Clin Monit Comput. 2016;30:55-61.

11. Aya HD, Rhodes A, Chis Ster I, Fletcher N, Grounds RM, Cecconi M. Hemodynamic effect of different doses of fluids for a fluid challenge: a quasi-randomized controlled study. Crit Care Med. 2017;45:e161-8.

12. Parkin G, Wright C, Bellomo R, Boyce N. Use of a mean systemic filling pressure analogue during the closed-loop control of fluid replacement in continuous hemodiafiltration. J Crit Care. 1994;9:124-33.

13. Cecconi M, Aya HD, Geisen M, Ebm C, Fletcher N, Grounds RM, et al. Changes in the mean systemic filling pressure during a fluid challenge in postsurgical intensive care patients. Intensive Care Med. 2013;39:1299-305.

14. Gupta K, Sondergaard S, Parkin G, Leaning M, Aneman A. Applying mean systemic filling pressure to assess the response to fluid boluses in cardiac post-surgical patients. Intensive Care Med. 2015;41:265-72.

15. Aya HD, Ster IC, Fletcher N, Grounds RM, Rhodes A, Cecconi M. Pharmacodynamic analysis of a fluid challenge. Crit Care Med. 2016;44:880-91.

16. Maas JJ, Pinsky MR, Geerts BF, de Wilde RB, Jansen JR. Estimation of mean systemic filling pressure in postoperative cardiac surgery patients with three methods. Intensive Care Med. 2012;38:1452-60.

17. Maas JJ, Pinsky MR, Aarts LP, Jansen JR. Bedside assessment of total systemic vascular compliance, stressed volume, and cardiac function curves in intensive care unit patients. Anesth Analg. 2012;115:880-7.

18. Wells G, Shea B, O'Connell J, Peterson J, Welch V, Losos M, et al: The Newcastle-Ottawa Scale (NOS) for assessing the quality of nonrandomised studies in meta-analyses. http://www​.ohri.ca/programs​/clinical_epidemiology/oxford.asp. 2009. Accessed 20 February 2017.

19. Liberati A, Altman DG, Tetzlaff J, Mulrow C, Gøtzsche PC, Ioannidis JP, et al. The PRISMA statement for reporting systematic reviews and meta-analyses of studies that evaluate health care interventions: explanation and elaboration. PLoS Med. 2009;6:e1000100

20. Moher D Liberati A, Telzlaff J, Altman DG, the PRISMA Group. Preferred Reporting Items for Systematic Reviews and Meta-Analyses: The PRISMA statement. PLoS Med.2009;6;e1000097
